# Detection of SARS-CoV-2 N protein using AgNPs-modified aligned silicon nanowires BioSERS chip†

**DOI:** 10.1039/d4ra00267a

**Published:** 2024-04-16

**Authors:** Sadok Kouz, Amal Raouafi, Awatef Ouhibi, Nathalie Lorrain, Makram Essafi, Manel Mejri, Noureddine Raouafi, Adel Moadhen, Mohammed Guendouz

**Affiliations:** a Faculty of Sciences of Tunis, Laboratory of Nanomaterials Nanotechnology and Energy (L2NE), University of Tunis El Manar 2092 Tunis El Manar Tunisia adel.modhen@istmt.utm.tn; b UMR FOTON, CNRS, University of Rennes Enssat, BP 80518, 6 rue Kerampont F22305 Lannion France mohammed.guendouz@univ-rennes.fr; c Faculty of Sciences of Tunis, Laboratory of Analytical Chemistry and Electrochemistry (LR99ES15), Sensor and Biosensors Group, University of Tunis El Manar 2092 Tunis El Manar Tunisia noureddine.raouafi@fst.utm.tn; d Pasteur Institute of Tunis, University of Tunis El Manar LTCII LR11 IPT02 Tunis Tunisia

## Abstract

The SARS-CoV-2 (COVID-19) pandemic had a strong impact on societies and economies worldwide and tests for high-performance detection of SARS-CoV-2 biomarkers are still needed for potential future outbreaks of the disease. In this paper, we present the different steps for the design of an aptamer-based surface-enhanced Raman scattering (BioSERS) sensing chip capable of detecting the coronavirus nucleocapsid protein (N protein) in spiked phosphate-buffered solutions and real samples of human blood serum. Optimization of the preparation steps in terms of the aptamer concentration used for the functionalization of the silver nanoparticles, time for affixing the aptamer, incubation time with target protein, and insulation of the silver active surface with cysteamine, led to a sensitive BioSERS chip, which was able to detect the N protein in the range from 1 to 75 ng mL^−1^ in spiked phosphate-buffered solutions with a detection limit of 1 ng mL^−1^ within 30 min. Furthermore, the BioSERS chip was used to detect the target protein in scarcely spiked human serum. This study demonstrates the possibility of a clinical application that can improve the detection limit and accuracy of the currently commercialized SARS-CoV-2 immunodiagnostic kit. Additionally, the system is modular and can be applied to detect other proteins by only changing the aptamer.

## Abstract 2

A sensitive and selective bioSERS chip was developed for the detection of nucleocapsid protein from SARS-CoV-2 virus, applied to sense the N protein in human blood serum.

## Graphical abstract

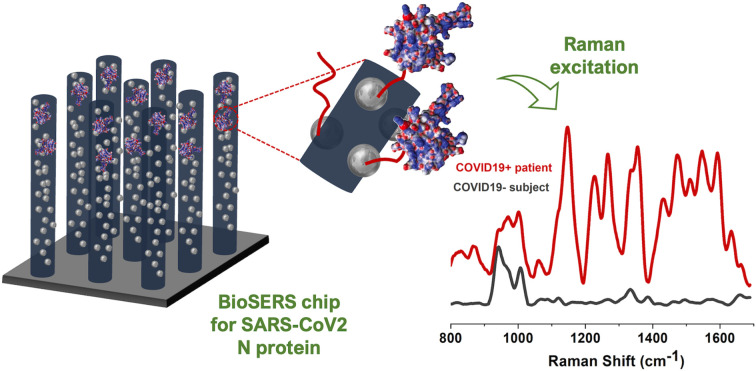

## 1. Introduction

In late 2019, a novel severe acute respiratory syndrome (SARS-CoV-2) virus of unknown origin appeared in Wuhan (China).^1^ SARS-CoV-2 affects the lungs damaging them severely and often causing lung failure,^2^ leading to death. To date, the worldwide toll of death is around 6.98 million, but it could be much higher since all the deaths were not reported. This new coronavirus caused the disease of COVID-19 around the world leading to a global pandemic.^3^ The SARS-CoV-2 infection rate reached alarming levels due to its easy transmission through direct contact *via* respiratory droplets from an infected person or by touching surfaces contaminated with the virus.^4,5^ Following its discovery, countries worldwide implemented strict safety measures, such as border closures, mandatory self-quarantine, and the shutdown of public and crowded spaces. These measures have significantly impacted global economic growth, leading to the bankruptcy of numerous businesses. This crisis has had a devastating effect on the global economy, resulting in losses exceeding $16 trillion.^6^ This high rate of transmission has prompted scientists and researchers worldwide to focus on developing new detection methods alternative to cumbersome real-time polymerase chain reaction or modifying existing ones to effectively identify the virus in its early stages of development. Detecting the virus at early stages can help mitigate its widespread impact.

Besides 16 non-structural proteins (NSPs1-16),^7^ the main four proteins of the novel coronavirus are the spike (S), the membrane protein (M), the envelope protein (E), and the nucleocapsid protein (N), which is the only core protein. The latter is the most immunogenic one triggering a high immune response among infected individuals.^8^ The N protein is well studied in SARS-CoV-1 (ref. 9) and it is associated with the viral RNA function and plays a main role in viral transcription.^10^ While the S protein is currently the primary target for COVID-19 biosensing applications due to its indispensable functions, recent studies have shown that in some cases the N protein is more sensitive than the S protein for detecting early infection.^11^ Moreover, the latter is subject to several mutations in the RBD zone such as K417N, S477N, E484A, Q493K, G496S, Q498R, *etc.*, which will require preparing a specific detector for each mutant. Thus, targeting the N protein seems a more forward approach for COVID-19 detection in sera and nasal swabs using rapid antigenic tests.

Aptamer-based biosensors employ aptamers as recognition elements linked to a physical signal transducer, translating the binding interaction between the aptamer and its target into a detectable signal, thereby facilitating precise and selective identification of particular analytes.^12,13^ The use of aptamers, which are single-stranded DNA or RNA oligonucleotides, in biosensing as specific receptors for quick screening of disease biomarkers has garnered significant interest. To replace antibodies, which are often costly and subject to degradation, aptamer-based biosensing devices are used.^14,15^ When it comes to early screening of disease, aptamer-based techniques have several advantages over conventional antibody assays.^16–19^ Their primary advantages are high selectivity, affinity, and stability, which enable them to distinguish between protein isoforms and splice variants and increase the potential for numerous applications, even in multiplex discovery platforms.^20^ Additionally, the longevity of a commercial aptamer-based detection kit is increased by the stability of aptamers, which can even be regenerated after denaturation.

Among several sophisticated analytical instruments, surface-enhanced Raman spectroscopy is a powerful analytical tool that can be used in the detection of different biological molecules.^21^ SERS can provide a precise fingerprint spectrum even in complex samples with very low concentrations. Also, it is a non-destructive method that needs a short analysis time and provides a high sensitivity and low detection limit. In recent work, we have successfully designed a SERS biosensor to detect prostate-specific antigens using an aptamer tethered to silicon nanowires (SiNWs) decorated with silver nanoparticles (AgNPs).^22^ Lysozyme is a food allergen that could contaminate food during the processing step. Boushell *et al.* designed a SERS-based sensor for the detection of lysozyme on food-handling surfaces.^23^ Negri *et al.* reported the design of two different SERS platforms for the detection of Influenza A through the detection of its nucleoprotein using an aptamer.^24,25^ Chen *et al.* reported the SERS detection of the whole Influenza A/H1N1 virus using an aptamer targeting the viral hemagglutinin surface protein and popcorn-like gold nanostructures for signal enhancement.^26^

In this work, we developed a SERS-based aptasensor platform capable of detecting the SARS-CoV-2 N protein with a high sensitivity. A specific DNA aptamer for the N protein is used as a receptor and an AgNPs/SiNWs substrate for signal transducing. The SiNWs fabrication and their decoration with AgNPs are achieved by a relatively simple wet-lab chemical processing method.^22^ A quantitative analysis of the SARS-CoV-2 lysate is performed by monitoring the change in the SERS peaks intensity and area caused by the new binding between the aptamer DNA attached to the AgNPs/SiNWs surface and the N protein in the SARS-CoV-2 virion. This biosensor enables detecting the target with a limit of detection of 1 ng mL^−1^ in less than 30 min. This methodology is costly effective, robust, rapid, and requires very small sample volumes, which can help in the diagnosis domain of infections at very early stages.

## 2. Methods

### 2.1. Chemicals and reagents

Silver nitrate (>99%), hydrofluoric acid (50%), hydrogen peroxide (34%), nitric acid (70%), sulfuric acid (97%), 6-mercaptohexanol (MCH), and cysteamine hydrochloride were purchased from Sigma-Aldrich (Germany). All reagents were of analytical grade and used without further purification. Phosphate-buffered saline solution (PBS, 10 mM) was prepared by dissolving the required amounts of KCl, NaCl, K_2_HPO_4_, and KH_2_PO_4_ in deionized water.

All the solutions were prepared using deionized water produced by a Millipore system. The 5′-thiolated anti-N specific DNA aptamer (5′-aaa aac gcg cgt att cct tag ggg cac cgc tac acg cgc g-3′) was acquired from Biomers (Germany). The sequence was purified by HPLC to ensure the maximum purity and the reproducibility of the test. This DNA aptamer was recently discovered by Zhang *et al.* for the targeting of recombinant COVID-19 nucleocapsid protein of SARS2-CoV-2.^27^ Membrane protein (M protein) and recombinant SARS-CoV-2 spike glycoprotein (S protein) were purchased from Abcam. The human blood serum used in this work was purchased from Sigma-Aldrich (product ref. H4522).

### 2.2. Production of a recombinant N protein

A His-tagged recombinant form of the COVID-19 N was expressed in *E. coli*. Briefly, the cDNA encoding for the COVID-19 N protein was first generated by reverse transcription using the Superscript II RT kit (Invitrogen). COVID-19 N encoding gene was later amplified using two primers, NF1 5′-cagggattccgatgtctgataatggaccccaaa-3′ and NR1 5′-ctcgtcgacggcttgagttgagtcagcactgc-3′. The PCR product was later digested with BamH1 and Sal1 restriction enzymes and ligated to the pET26 vector, linearized by BamH1 and Xho1 enzymes. A recombinant construct was later used to transform the BL21 *E. coli* strain for protein expression and purification in native conditions. The recombinant protein was first purified on a Nickel-Sepharose column and then on a G75 Sepharose gel filtration column. Protein purity reached more than 95%, as assessed on SDS-PAGE gel.

### 2.3. Instrumentation

Raman measurements were performed on a Micro Raman HORIBA system (LabRAM HR800) at room temperature (RT) with a helium–neon laser (*λ* = 632.8 nm) and a power of 2.4 mW at the sample surface, with a 100× microscope objective.

The morphological properties of the surface and the cross-section of the samples were analyzed using a JEOL JSM 7100F thermal field emission electron gun scanning electron microscope (SEM). Composition analysis of the cross-section of the samples was performed using a conventional HITACHI FLEX SEM II microscope equipped with an energy-dispersive detector (EDX).

The absorbance spectra were obtained at RT in the wavelength range of 250 to 400 nm using an UV-Visible-NIR spectrophotometer (PerkinElmer Lambda 950).

### 2.4. Elaboration of the BioSERS platform

**Fig. 1:**
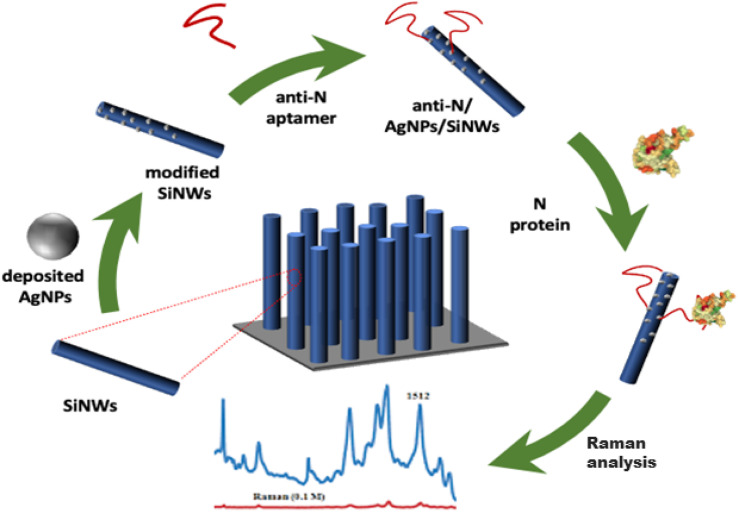
Schematic illustration of the step-by-step preparation of the BioSERS sensing chip and detection of the N protein.

The design of the BioSERS platform for detecting the N protein involved a series of sequential steps. Initially, SiNWs were fabricated in 2 steps using a metal-assisted chemical etching technique. This involved treating lightly N-doped Si (100) wafers with an HF solution.^28^ To deposit AgNPs onto the SiNWs, we employed the chemical reduction of Ag^+^ using Si–H groups. The optimization of these parameters had already been carried out in a previous work.^22^ To prevent the non-specific binding of analytes to the silver surface, a critical step in our protocol involved the strategic use of a self-assembled monolayer (SAM). In our previous work,^22^ we employed MCH as a SAM, and this was applied before introducing the aptamer onto the SERS substrate. However, in the current study, we introduced a key modification. Instead of applying the SAM before the aptamer, we opted for a different SAM composed of cysteamine, and this step was performed after the attachment of the aptamer. This decision was driven by a two-fold consideration. Firstly, cysteamine, with its unique molecular structure, served as an effective blocking agent, preventing non-specific adsorption of analytes onto the bare silver surface.^29^ Secondly, placing the cysteamine SAM after the aptamer attachment underscored the adaptability and versatility of our SERS substrate. By immersing the substrate in a solution of cysteamine hydrochloride after the aptamer was in place, we ensured that the SAM formation precisely complemented the functionalization with the aptamer. This sequence of steps was carefully designed to optimize the performance of our aptasensor. To achieve this, the thiolated DNA aptamer was tethered to the surface of the AgNPs by incubating the sample into a 0.5 mL solution containing 1 μM of anti-N aptamer dissolved in PBS for 4, 8, 12, and 16 hours at RT. Next, we immersed the SERS substrate in a solution of 10^−3^ M cysteamine hydrochloride dissolved in 10 mM PBS solution.^30^ Subsequently, the sample was removed from the solution, washed twice with PBS and deionized water, and dried under a nitrogen flow. In the final stage, the aptasensor (cysteamine/anti-N/AgNPs/SiNWs) underwent incubation in different concentrations of N protein dissolved in a PBS solution at a pH of 7.4. The incubation time for this step ranged from 20 to 30 minutes.^31^ Finally, the prepared substrates were thoroughly washed with deionized water and dried before Raman measurement. Fig. 1 schematically depicts the preparation of the BioSERS chip.

## 3. Results

### 3.1. Surface characterizations

**Fig. 2:**
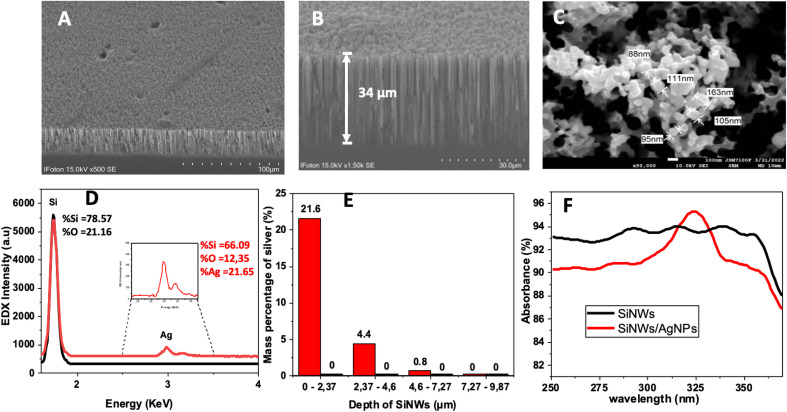
SEM images of (A) and (B) cross-section image of SiNWs and (C) surface top view of the AgNPs/SiNWs. (D) EDX Intensity spectra of SiNWs (black) and AgNPs/SiNWs (red) at the surface of the substrate (0–2 μm). (E) Mass percentage of silver *versus* depth of AgNPs/SiNWs substrate. (F) UV-visible absorbance spectra of SiNWs (black) and AgNPs/SiNWs (red).

In the context of SERS, the uniform distribution of metallic nanoparticles on the substrate plays a pivotal role in determining the reliability and reproducibility of the measurements. Fig. 2A–C provide insights into the surface morphology of the AgNPs/SiNWs substrate, showcasing both the cross-sectional and top-view perspectives. The uniformity of the material is of paramount importance for SERS efficacy. In SERS, the enhancement of Raman signals is highly dependent on the localized surface plasmon resonance (LSPR) generated by metallic nanoparticles. The spatial arrangement and density of these nanoparticles influence the “hot spots” on the substrate, which are regions of intensified electromagnetic fields. These hot spots are crucial for enhancing the interaction between the incident light and the molecules of interest, leading to enhanced Raman signals. Uniform distribution ensures consistent hot spot formation across the substrate, contributing to the reproducibility of SERS measurements. Deviations in distribution could result in uneven enhancement factors, affecting the intensity and reliability of the obtained spectra. Therefore, the observed homogeneity in the AgNPs/SiNWs material is a critical factor in achieving reliable and reproducible SERS results. During the fabrication process, several meticulous steps were taken to ensure the uniform distribution of AgNPs. Additional SEM images, captured at a lower magnification (×5), were included to provide a broader view of the substrate's surface, emphasizing the distribution pattern of the metallic nanoparticles. Moreover, the optimization of parameters during the deposition of AgNPs onto SiNWs and the subsequent functionalization steps with the thiolated DNA aptamer were carefully tuned to promote uniform coverage. The success of the uniform distribution is evident in both SEM images and EDX spectra, confirming the penetration of AgNPs into the pores of the SiNWs and the consistent coverage over the surface. These measures not only enhance the reliability of SERS measurements for the detection of the N protein but also underscore the adaptability and versatility of the developed SERS substrate.

To ascertain the functionalization with silver nanostructures, elemental composition by EDX (Fig. 2D and E) and UV-visible absorbance (Fig. 2F) spectroscopies were carried out before and after treatment of the silver nanoparticles. The UV-visible absorbance spectrum of the metalized substrate shows a peak around 320 cm^−1^ attributed to the surface plasmons due to the presence of the silver nanoparticles. The EDX elemental analysis evidenced the presence of AgNPs and showed the penetration of the metallic nanoparticles into the pores of the SiNWs. One can see from the results, the presence of % Si = 66.09, % O = 12.35, and % Ag = 21.65. The presence of new peaks at energy above 3 keV related to Lα emission of silver, confirms the success of the introduction of the AgNPs. The EDX spectrum performed on the cross-section indicated that the silver mass percentage from the surface to a depth of 2.37 μm was approximately 21%. Then, the percentage gradually decreases as the depth increases becoming zero for depths beyond 4.6 μm. The results provide evidence that the silver nanoparticles successfully modified the silicon nanowires.

**Fig. 3:**
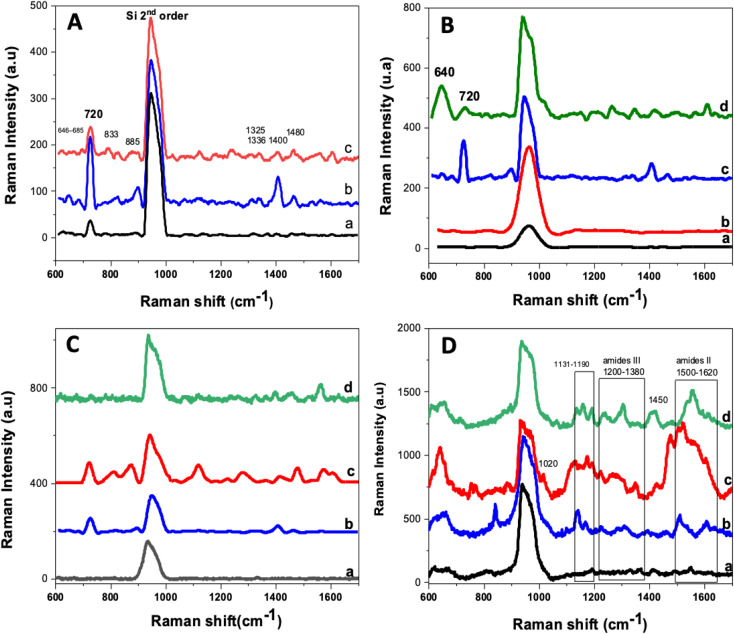
Raman spectra of (A) anti-N/AgNPs/SiNWs incubated for 4 hours in different concentrations of aptamer: ((a) 0.5 μM, (b) 1 μM, (c) 5 μM), (B) the (a) bare SiNWs, (b) AgNPs-modified SiNWs, (c) aptamer/AgNPs/SiNWs and (d) cysteamine/anti-N/AgNPs/SiNWs incubated for 4 hours in 1 μM solution of the aptamer, (C) different incubation times of aptamer: 2 h (a), 4 h (b), 8 h (c) and 16 h (d) of anti-N (1 μM)/AgNPs/SiNWs and (D) detection of N protein (15 ng mL^−1^, 30 min) at different incubation times of aptamer (1 μM): (a) 2 hours, (b) 4 hours, (c) 8 hours, and (d) 16 hours in the presence of cysteamine.

Raman spectroscopy was employed to further confirm these modifications. Specifically, the overlap of the spectra obtained for the silicon nanowires at each modification step indicates the existence of organic groups derived from the aptamer and the cysteamine-blocking group (Fig. 3).

### 3.2. Optimization of the parameters

#### 3.2.1. Aptamer concentration

Several key parameters are expected to affect the response of the BioSERS chip such as the incubation time of aptamer with the AgNPs/SiNWs substrate or the time necessary for the binding of target to the aptamer receptor. Since the functionalization of the surface of silicon nanowires with the aptamer is important for the detection step and to achieve high sensitivity, we choose to incubate the AgNPs/SiNWs substrate in a rather large amount (0.5 mL of 0.5 μM, 1.0 μM or 5.0 μM) of the aptamer solution for 4 hours of incubation using the Raman signature of the N protein as a probe to assess the sensitivity to it.

Fig. 3A depicted the different Raman spectra, revealing the emergence of novel modes subsequent to incubating the AgNPs/SiNWs substrate in different concentrations of the aptamer solution. The presence of new SERS modes at 500–800 cm^−1^, 800–1200 cm^−1^, and 1200–1600 cm^−1^ alongside the second-order silicon mode at 960 cm^−1^ indicates the successful tethering of the aptamer on the surface of silver nanoparticles. Upon careful comparison of the intensity of the modes for the three concentrations, it is evident that the medium concentration yielded the most distinct spectrum. Furthermore, the Raman spectra obtained at different aptamer concentrations revealed an interesting non-linear response. Specifically, lower intensity peaks were observed for the 0.5 μM concentration, which increased at 1 μM, and then decreased for 5 μM. This non-linear response may be influenced by factors such as the formation of a thicker layer of analyte at higher concentrations, impacting molecular orientation and interaction with the metallic surface. Similar observations have been reported in previous studies, where the presence of a thick layer of analyte molecules hindered the SERS effect, leading to decreased signal intensities. For instance, in a study by Yang *et al.*,^32^ it was found that at micromolar concentrations, the SERS effect was hindered by a thick layer of analyte molecules formed on the surface, potentially resulting in bulk Raman scattering rather than SERS. To address this issue, the gold surface was backfilled with a self-assembled monolayer of hexanethiol immediately after aptamer immobilization. This SAM prevents nonspecific adsorption of analyte molecules by blocking access to the surface and improves the efficiency of aptamer capturing. We therefore used the 1 μM concentration for the rest of our study.

#### 3.2.2. Filling agent addition

We further added cysteamine to block the surface and prevent the non-specific adsorption of the target on the bare surface of AgNPs. The modified surface by cysteamine (10^−3^ M for 30 minutes) was evaluated by Raman measurements. Spectrum (d) in Fig. 3B showed the appearance of a new mode related to cysteamine with very high intensity at 640 cm^−1^ which is attributed to the C–S bond.^33^ Furthermore, the main modes of the aptamer decreased in their scattered intensities such as the mode at 720 cm^−1^. The slight intensity decrease of aptamer vibration modes can be explained by the exchanging of the aptamer with the cysteamine and also the concentration difference between the two of them (10^−3^ M for cysteamine *vs.* 1 μM for aptamer).

#### 3.2.3. Incubation time of the aptamer

The AgNPs/SiNWs were immersed in an aptamer solution (1 μM) for different incubation times (4, 8, 12 and 16 h). The SERS spectra recorded from 600 to 1700 cm^−1^ is shown in Fig. 3C.

Due to its composition as a single-stranded DNA, the aptamer displays distinct and prominent Raman signatures that are typical of oligonucleotides. The identification and interpretation of these spectral bands were established through different research and analysis.^34–36^ In general, the Raman spectrum of DNA is divided into three regions that correspond to specific vibrational modes, including the vibrations of the DNA bases (500–800 cm^−1^), phosphate and sugar (800–1200 cm^−1^), and finally those of the DNA skeleton (1200–1600 cm^−1^), which are particularly influenced by the secondary structure of DNA.^35^ In particular, the main vibrational modes detected for the aptamer are outlined in Table 1.

**Table 1:** Assignment of the SERS peaks of the aptamer and SARS COV2 N protein

Cmpd	Raman shifts (cm^−1^)	Assignments	Ref.
Aptamer	720	Adenine: ring stretching	34
	646–685	In-ring breathing mode of guanine	35
	833	Thymine: C–C and C–N stretching	34
	885	Sugar vibration (C3′ *endo*)	36
	1325	Deoxyguanosine, C2′ *endo*/*syn*	36
	1336	Skeletal ring-vibration mode	35
	1376	Deoxythymidine	36
	1400	Deoxyribosyl C_5_H_2_ deformation	36
	1480	C_8_=N_7_–H_2_ deformation	36
Protein	1020	N–H deformation in deoxyguanosine	38
	1131–1190	Nitrogenous bases of the DNA nucleotides	35, 38 and 39
	1200–1380	Amides III	39–41
	1400–1480	CH_2_ and CH_3_ deformation in proteins	39 and 40
	1500–1620	Amides II	39–41

For longer incubation durations, the SERS spectra showed that the intensity of peaks increased but we also lost resolution since the signal width increased as well, probably due to a steric hindrance and the presence of a large amount of DNA strands on the surface of silver nanoparticles. The observed disappearance of the signal after 16 hours of incubation is attributed to the high and excessive adsorption of the aptamer on the surface of the nanoparticles. Prolonged incubation times can lead to saturation and overcrowding of the surface.^37^ This phenomenon, while influencing the SERS signal strength, also highlights the importance of optimizing incubation times for achieving a balance between effective functionalization and maintaining signal stability. Hence, incubation for 8 hours showed the best result and was selected further to carry out the rest of the work.

DNA mode observed at 720 cm^−1^, attributed to adenine ring stretching (Table 1), exhibited a significantly high intensity and distinguished itself from the others. Additionally, it is not included in the amide II and amide III regions so it can be used to assess the binding of the target protein, which will appear in the region of 1100 cm^−1^ to 1650 cm^−1^.

#### 3.2.4. Protein recognition

Since the amount of the target protein tethered to the surface of the BioSERS chip is dependent on the aptamer quantity present on the surface of AgNPs, we examined the effect of the aptamer incubation time (2, 4, 8, and 16 h) on the N protein. Also, it was intended to verify whether the duration of 8 h, arbitrarily posed in the preliminary experiments, was the adequate one. Fig. 3D showed the SERS spectra of N protein (15 ng mL^−1^) incubated with the bioSERS chip for different durations. For short incubation times (2 and 4 h), the spectra do not clearly show the stretching modes of the target analyte. For longer incubation times, Raman vibration modes of amides are visible, indicating the presence of the N protein.

As we can see, the peaks related to cysteamine (615–680) cm^−1^ were still visible, which confirmed that the surface is still blocked, the observed peaks are due to the target protein bound to the aptamer. Furthermore, new intense peaks appear at 1020 and 1400 to 1480 cm^−1^ and in the ranges of 1131 to 1190 cm^−1^, 1200 to 1380 cm^−1^ and 1500 to 1620 cm^−1^. The peaks at 1020 cm^−1^ and the peaks in the range of 1400–1480 cm^−1^ range are related to N–H deformation in deoxyguanosine, and CH_2_ and CH_3_ deformation in proteins, respectively.^38–40^ The peaks in the 1131–1190 cm^−1^ range are related to the nitrogenous bases of the DNA nucleotides. Indeed, the 1087 cm^−1^ mode is related to the *ν*_CO_ stretching in amides and proteins, the 1136 cm^−1^ mode is assigned to the *ν*_NH_2__ of the guanine, the 1180 cm^−1^ mode is attributed to deoxythymidine, the 1173 and 1176 cm^−1^ modes represent the *ν*_CH_ of the tyrosine.^35,38,39^

The most specific peaks are located in the ranges 1200 to 1380 cm^−1^ and 1500 to 1620 cm^−1^ which are assigned to the *ν*_CN_ and *ν*_NH_ modes of amides III and II, respectively.^39–41^ The intensities of these modes increased monotonously upon time increased from 2 h to 8 h and then decreased for 16 h. The optimal incubation time of AgNPs/SiNWs in aptamer was set to 8 h to carry out the experiment since it has shown the best results both for aptamer/AgNPs/SiNWs and N-protein/cysteamine/aptamer/AgNPs/SiNWs.

### 3.3. Performance of the BioSERS chip

**Fig. 4:**
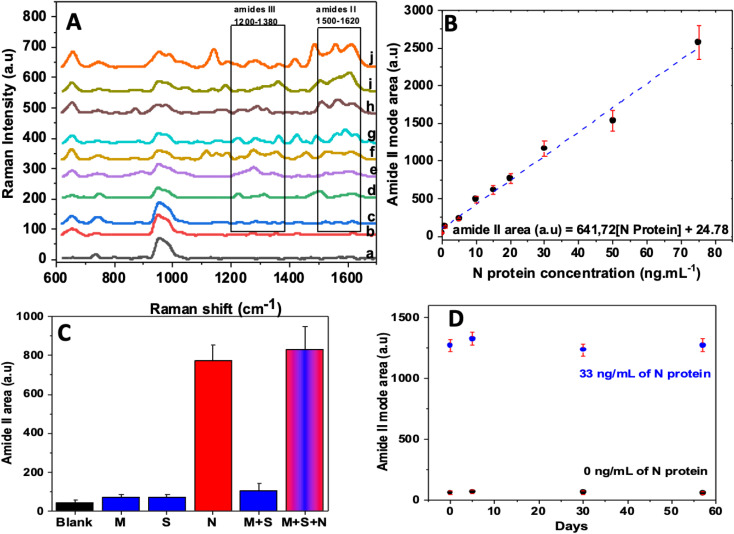
(A) SERS spectra of bioSERS chip aptamer/cysteamine/AgNPs/SiNWs in the presence of different protein concentrations 1 ng mL^−1^ (c), 5 ng mL^−1^ (d), 10 ng mL^−1^ (e), 15 ng mL^−1^ (f), 20 ng mL^−1^ (j), 30 ng mL^−1^ (h), 50 ng mL^−1^ (i) and 75 ng mL^−1^ (j), (B) calibration plot displaying the amide II mode (1500 to 1630 cm^−1^) area *versus* the concentration of the target protein. (C) Analytical response of the aptasensor toward different proteins: N (33 ng mL^−1^), S (33 ng mL^−1^) and M (33 ng mL^−1^); amide II mode area for each type of analyte. (D) Storage stability of the aptasensor stored for 57 days at 4 °C and its response in presence of 33 ng mL^−1^ of N protein recorded periodically.

Fig. 4A displayed the SERS spectra, before and after adding the target protein to the cysteamine/aptamer (8 h)/AgNPs/SiNWs bioSERS chip. The N protein concentrations ranged from 1 to 75 ng L^−1^. The protein addition induced the appearance of new peaks, which increased with the target increasing concentrations. The latter indicated that the amount of N protein bound to the aptamer is becoming important. The main vibration modes are the amide III in the range 1200–1380 cm^−1^ and the amide II in the range 1500–1620 cm^−1^. The peak area of the amide II vibration located at the range of 1500–1620 cm^−1^ was used to plot the calibration as a function of the concentration (Fig. 4B). The calibration plot is linear in the range from 1 to 75 ng mL^−1^, following eqn (1):

1 Amide II area (a.u.) = 641.72[N protein] (ng mL^−1^) + 24.78

The correlation coefficient of the linear curve is *R*^2^ = 0.989 and the limit of detection is estimated to be the lowest detection concentration 1 ng mL^−1^, calculated from the 3 × *S*_b_/*m* criterion, where *m* is the slope of the calibration curve and *S*_b_ was estimated as the standard deviation of three different measurements recorded for the lowest analyte concentration.

### 3.4. Selectivity and shelf-life

#### 3.4.1. Selectivity and specificity

Selectivity and specificity play a crucial role in determining the performance and reliability of any detection method. To evaluate these important characteristics, we considered the S protein and M protein of the coronavirus as competing proteins for the target protein. The selectivity and specificity tests were carried out under identical conditions to that used the detect the target protein, where each protein (S, M, and N) was added at a concentration of 33 ng mL^−1^.

In Fig. 4C, the peak area of amide II for each type of protein was plotted. It can be observed that a distinctive difference in the intensity of peaks within the BioSERS chip was exposed to 33 ng mL^−1^ of different examined proteins. Specifically, the N protein led to a noticeable increase in SERS peak intensity for the modes associated with proteins, whereas the S and M proteins did not cause any significant changes. This outcome demonstrates the exceptional selectivity of the biosensor towards the N protein. Furthermore, to assess the specificity of the system, the response of the chip in the presence of the two aforementioned proteins in the presence and absence of the N protein was examined. Interestingly, no significant response is obtained in the absence of the N protein. This finding provided further evidence of the chip's ability to specifically detect the N protein while maintaining minimal interference from other proteins.

#### 3.4.2. Shelf-life

To assess the storage stability of the bioSERS chip, it was carefully monitored approximately for two months when it was stored in a refrigerator at 4 °C. During this time, response was periodically recorded both with and without the presence of the N protein. As depicted in Fig. 4D, the chip showcased a consistent and stable signal even after 57 days of storage (longer durations were not assayed), whether in the absence or presence of 33 ng mL^−1^ of N protein.

### 3.5. Applicability in complex matrix

**Fig. 5:**
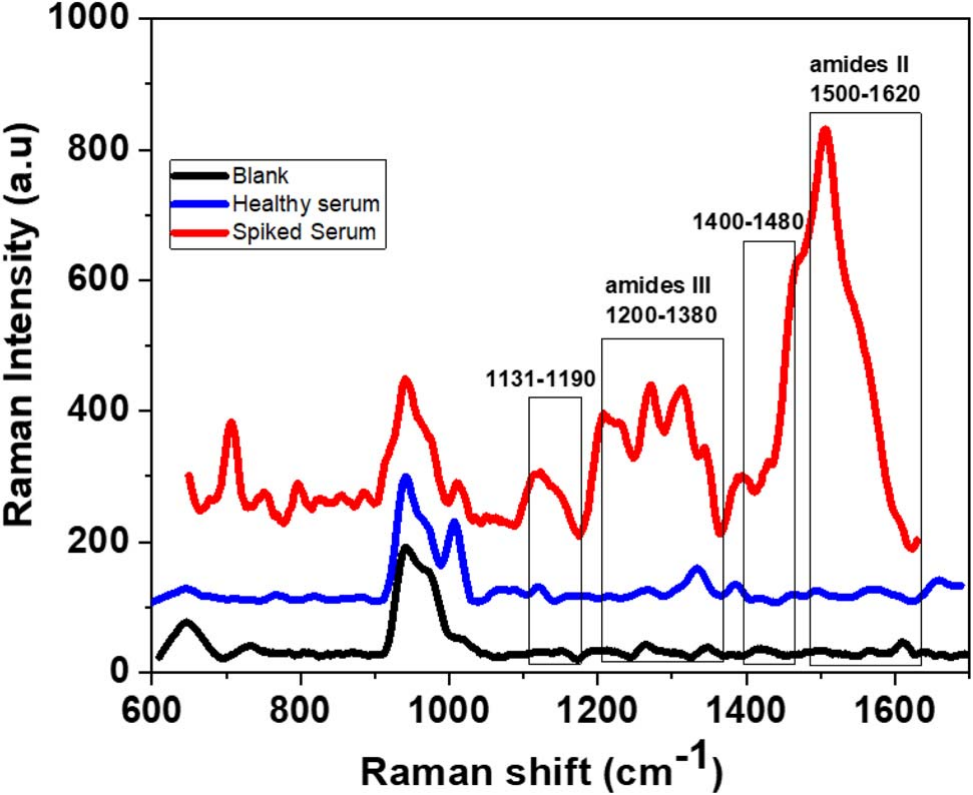
BioSERS response to the healthy blood serum before and after the introduction of N protein into the mixture.

To better assess the selectivity of the chip, its performance was tested on complex samples, specifically using human blood serum from a healthy donor. The serum contains an assortment of compounds including proteins and peptides like albumins, globulins, lipoproteins, enzymes, and hormones. Additionally, vital nutrients such as carbohydrates, lipids, and amino acids are present, as well as electrolytes, organic waste, and a variety of small organic molecules, both suspended and dissolved. In this study, 150 ng mL^−1^ of N protein was introduced into the 10×-diluted serum with phosphate buffer saline solution to decrease its viscosity. Using the same detection procedure employed with the buffered solutions, Fig. 5 illustrated the response in the presence of the diluted serum, as well as after the addition of N protein to the mixture.

Particularly amide II group is visible for the spiked serum sample while it is completely absent for the diluted serum of the healthy subject. This finding highlights the ability of the BioSERS chip to distinguish and detect the N protein even in complex biological samples such as blood serum.

### 3.6. Comparison with other works

The BioSERS chip developed for the N protein detection exhibited superior analytical performance compared to previously reported works, as indicated in Table 2. Although its detection limits and dynamic ranges are slightly lower than those of the electrochemical and colorimetric biosensors reported in ref. 42 and 43, and its detection time is shorter than that of SERS biosensors mentioned in ref. 44–46, this device offers the advantage of being easier to manufacture and having a longer shelf life of up to 2 months. Additionally, it outperforms in terms of detection limit the biosensors utilizing calorimetric assay and SERS spectroscopy, as well as some of the electrochemical biosensors mentioned in ref. 44–48.

**Table 2:** Comparison of different detection strategies for SARS-CoV-2 virus^a^

Biosensor	Target	Biological sample	DL (ng mL^−1^)	Time (min)	Ref.
Calorimetric assay (AuNPS-60 nm)	N-Gene from isolated RNA	Vero cells infected with SARS-CoV-2	180	10	47
Paper based electrochemical biosensor	SARS-CoV-2 antigen & antibodies	Serum sample	0.11	—	42
Electrochemical sensor	S-Protein	Untreated saliva	19	—	48
SERS (ACE2 with AuNPs)	S-Protein	Contaminate water & simulated urine sample	80^b^	5	44
SERS (MXenes)	S-Protein	NT	5^c^	10	45
SERS (Au–Cu nano stars)	S-Protein	NT	8.89^c^	15	46
Colorimetric/SERS/fluorescence triple mode biosensor (AuNPs)	Viral RNA	NT	0.16^c^	40	43
SERS (AgNPs)	Lysozymes	Egg allergen	500	40	23
SERS (Ag nanorod)	Influenza N-protein	NT	10^6^	8^e^	24
SERS (Ag nanorod)	Influenza N-protein	Cell lysates	10^3^	8^e^	25
SERS (Au nano-popcorn)	A/H1N1	NT	97^d^	20	26
SERS (SAM of Au-NPs)	SARS-CoV-2 target gene	Target DNA solutions	3.1 × 10^−15^^f^	60	49
SERS (AgNPs/SiNWs)	N-Protein	Contaminate blood serum	1	20–30	This work

a NT: not tested.

b Copies per mL.

c nmol L^−1^.

d PFU mL^−1^.

e Hours.

f Molar concentration.

## 4. Discussion

The paper reports the design and characterization of a BioSERS chip for the detection of the N protein, a key component of the coronavirus-2, which originated the COVID-19 pandemic in 2019. The study involves several crucial steps, including surface characterizations, optimization of operational parameters, performance evaluation, selectivity and specificity testing, shelf-life assessment, and applicability in spiked buffered solutions, complex matrices, and real samples.

The surface characterizations demonstrated the successful modification of silicon nanowires with silver nanoparticles and the anti-nucleocapsid aptamer. The consistent and uniform structure of SiNWs, the spherical shape of AgNPs, and the confirmation of the successful introduction of AgNPs through SEM images, EDX analysis, and UV-visible spectroscopy provided a strong foundation for the subsequent experiments. Raman spectroscopy further confirmed the modifications, indicating the presence of the signature of the organic groups derived from the aptamer building blocks and cysteamine-blocking agent.

We further examined various parameters to optimize the sensitivity of the BioSERS chip. Indeed, the aptamer concentration testing revealed that a medium concentration (1 μM) yielded the most distinct spectrum, indicating successful aptamer tethering. The addition of cysteamine was beneficial since it efficiently blocked the surface to prevent the non-specific adsorption of the target biomolecule, and an 8 hours incubation period for the aptamer was determined as the optimal duration. This duration provided a balance between signal intensity and resolution, making it suitable for further experiments.

The BioSERS chip demonstrated excellent performance in detecting the nucleoprotein. The calibration plot shows a linear relationship between the amide II peak area and N protein concentration varying from 1 to 75 ng mL^−1^, with a low detection limit of 1 ng mL^−1^. Selectivity and specificity studies revealed that the device was able to selectively detect the N protein, distinguishing it from competing S and M proteins from the same virus and maintaining minimal interference. The chip also exhibited remarkable stability over a 57 days storage period. Longer storage durations were not assayed.

The applicability of the BioSERS chip in complex matrices was validated using spiked diluted serum, where it successfully detects the N protein even in the presence of various serum components. These results highlighted the device's potential for real-world applications, especially in clinical diagnostics and disease monitoring.

Comparative literature analysis showed that the developed BioSERS chip outperformed several previously reported sensing methods in terms of detection limit, ease of manufacturing, and shelf life. While some other methods exhibited slightly lower detection limits and wider dynamic ranges, the advantages of the BioSERS chip in terms of simplicity and longevity made it a promising candidate for practical use.

In addition to the excellent performance demonstrated by the BioSERS chip in detecting the nucleoprotein of SARS-CoV-2, further enhancements in sensitivity and detection capabilities can be achieved through the integration of surface-enhanced Raman spectroscopy with rolling circle amplification.^50^ The combination of these two techniques holds promise for amplifying the signal generated by the target biomolecule, thereby improving the chip's overall sensitivity, and lowering its limit of detection. Future research efforts could focus on integrating RCA amplification into the BioSERS chip platform to further enhance its sensitivity and broaden its applicability in detecting viral proteins like the N protein of COVID-19, even in complex biological matrices. This integrated approach would contribute to the ongoing efforts to develop highly sensitive and rapid diagnostic tools for combating infectious diseases.

## 5. Conclusion

The paper presents a comprehensive study on the development and characterization of a BioSERS chip for the sensitive and selective detection of the N protein. Its superior analytical performance and practical advantages position it as a valuable tool in the field of disease diagnostics and monitoring, particularly in the context of COVID-19. This technique enables detecting SARS-CoV-2 N protein with a detection limit of 1 ng mL^−1^ within 20 to 30 min. The findings pave the way for further research and potential commercialization of the developed BioSERS chip for widespread use in healthcare and related applications.

## Data availability

Data will be made available on request.

## Conflicts of interest

There are no conflicts to declare.

## Supplementary Material

RA-014-D4RA00267A-s001
