# Supplementary material for: Detection of SARS-CoV-2 N protein using AgNPs-modified aligned silicon nanowires BioSERS chip
Source: RSC Adv. 2024 Apr 16;14(17):12071–80. doi: 10.1039/d4ra00267a (PMC11019291; doi:10.1039/d4ra00267a)

Electronic Supplementary Material (ESI) for RSC Advances.
This journal is © The Royal Society of Chemistry 2024

Cloning and expression of a recombinant form of the Cov-2 N Protein in *E.Coli*

1- RT-PCR with N-specific primers

Used primers

NF1 5' cagGGATCCGatgtctgataatggaccccaaa 3' **BamHI**, for periplasmic expression

NR1 5' ctcGTCGACggcttgagttgagtcagcactgc 3' **Sal1** site

Digestion of the vector pET26 with BamH1 Xho1
Digestion of the Insert with BamH1 Sal1
Ligation (Xho1 and Sal1 are compatible)

```
      ATGAAATACCTGCTGCCGACCGCTGCTGCTGGTCTGCTGCTCCTCGCTGCCCAGCCGGCGATGGCC
      ATGGATATCGGAATTAATTCGGATCCGatgtctgataatggaccccaaaatcagcga
28321 aatgcaccccgcattacgtttggtggaccctcagattcaactggcagtaaccagaatgga
28381 gaacgcagtggggcgcgatcaaaacaacgtcggccccaaggtttacccaataatactgcg
28441 tcttggttcaccgctctcactcaacatggcaaggaagaccttaaattccctcgaggacaa
28501 ggcgttccaattaacaccaatagcagtccagatgaccaaattggctactaccgaagagct
28561 accagacgaattcgtggtggtgacggtaaaatgaaagatctcagtccaagatggtatttc
28621 tactacctaggaactgggccagaagctggacttccctatggtgctaacaaagacggcatc
28681 atatgggttgcaactgagggagccttgaatacaccaaaagatcacattggcacccgcaat
28741 cctgctaacaatgctgcaatcgtgctacaacttcctcaaggaacaacattgccaaaaggc
28801 ttctacgcagaagggagcagaggcggcagtcaagcctcttctcgttcctcatcacgtagt
28861 cgcaacagttcaagaaattcaactccaggcagcagtaggggaacttctcctgctagaatg
28921 gctggcaatggcggtgatgctgctcttgctttgctgctgcttgacagattgaaccagctt
28981 gagagcaaaatgtctggtaaaggccaacaacaacaaggccaaactgtcactaagaaatct
29041 gctgctgaggcttctaagaagcctcggcaaaaacgtactgccactaaagcatacaatgta
29101 acacaagctttcggcagacgtggtccagaacaaacccaaggaaattttggggaccaggaa
29161 ctaatcagacaaggaactgattacaaacattggccgcaaattgcacaatttgcccccagc
29221 gcttcagcgttcttcggaatgtcgcgcattggcatggaagtcacaccttcgggaacgtgg
29281 ttgacctacacaggtgccatcaaattggatgacaaagatccaaatttcaaagatcaagtc
29341 attttgctgaataagcatattgacgcatacaaaacattcccaccaacagagcctaaaaag
29401 gacaaaaagaagaaggctgatgaaactcaagccttaccgcagagacagaagaaacagcaa
29461 actgtgactcttcttcctgctgcagatttggatgatttctccaaacaattgcaacaatcc
29521 atgagcagtgctgactcaactcaagccGTCGAGCACCACCACCACCACCACTGA
```

Translate

Peptide signal PelB

MKYLLPTAAAGLLLLAAQPAMAMDIGINSDPMSDNGPQNQRNAPRITFGGPSDSTGSNQNGERSGARSKQRRP
QGLPNNTASWFTALTQHGKEDLKFPRGQGVPINTNSSPDDQIGYYRRATRRIRGGDGKMKDLSPRWYFYYLGTGP
EAGLPYGANKDGIIWVATEGALNTPKDHIGTRNPANNAAIVLQLPQGTTLPKGFYAEGSRGGSQASSRSSSRSRNSS
RNSTPGSSRGTSPARMAGNGGDAALALLLLDRLNQLESKMSGKGQQQQGQTVTKKSAAEASKKPRQKRTATKAY
NVTQAFGRRGPEQTQGNFGDQELIRQGTDYKHWPQIAQFAPSASAFFGMSRIGMEVTPSGTWLTYTGAIKLDDK
DPNFKDQVILLNKHIDAYKTFPPTEPKKDKKKKADETQALPQRQKKQQTVTLLPAADLDDFSKQLQQSMSSADSTQ
AVEHHHHHH

N protein as expressed in periplasmic area

Theoretical pI/Mw (average) for the user-entered sequence:

```
        10         20         30         40         50         60
MDIGINSDPM SDNGPQNQRN APRITFGGPS DSTGSNQNGE RSGARSKQRR PQGLPNNTAS

        70         80         90        100        110        120
WFTALTQHGK EDLKFPRGQG VPINTNSSPD DQIGYYRRAT RRIRGGDGKM KDLSPRWYFY

       130        140        150        160        170        180
YLGTGPEAGL PYGANKDGII WVATEGALNT PKDHIGTRNP ANNAAIVLQL PQGTTLPKGF

       190        200        210        220        230        240
YAEGSRGGSQ ASSRSSSRSR NSSRNSTPGS SRGTSPARMA GNGGDAALAL LLLDRLNQLE

       250        260        270        280        290        300
SKMSGKGQQQ QGQTVTKKSA AEASKKPRQK RTATKAYNVT QAFGRRGPEQ TQGNFGDQEL

       310        320        330        340        350        360
IRQGTDYKHW PQIAQFAPSA SAFFGMSRIG MEVTPSGTWL TYTGAIKLDD KDPNFKDQVI

       370        380        390        400        410        420
LLNKHIDAYK TFPPTEPKKD KKKKADETQA LPQRQKKQQT VTLLPAADLD DFSKQLQQSM

       430
SSADSTQAVE HHHHHH
```

Theoretical Mw: 47619.83 Da

Theoretical pI: 9.95

Purification of the recombinant N protein from the periplasmic space of BL21 on a Nickel Sepharose column

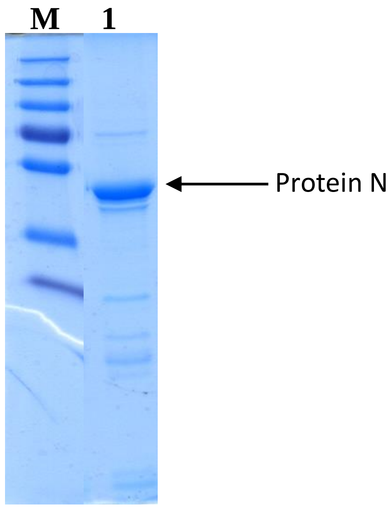

Purification of recombinant N on a Sepharose G50 column

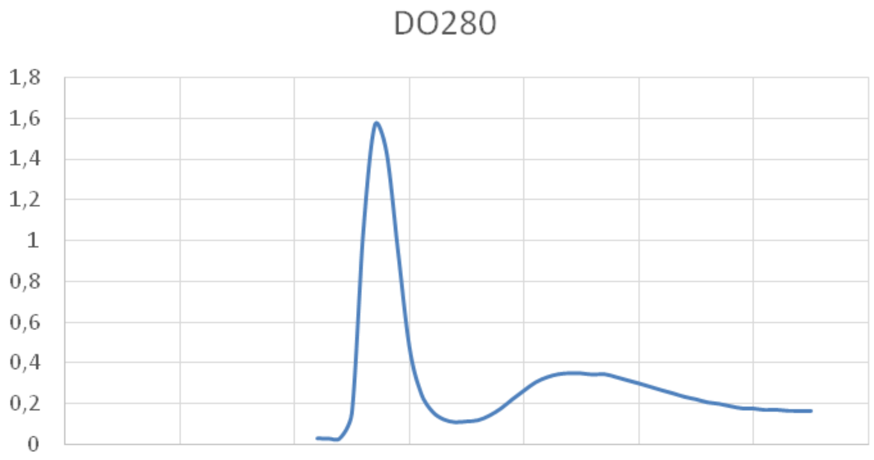

Analysis of the protein on SDS-PAGE gel

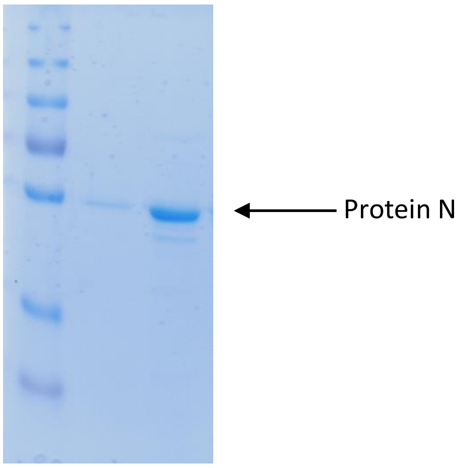

Supplement: RA-014-D4RA00267A-s001 [file RA-014-D4RA00267A-s001.pdf]
